# Response of MCF-7 Breast Cancer Cells Overexpressed with P-Glycoprotein to Apoptotic Induction after Photodynamic Therapy

**DOI:** 10.3390/molecules26237412

**Published:** 2021-12-06

**Authors:** Eric Chekwube Aniogo, Blassan P. George, Heidi Abrahamse

**Affiliations:** Laser Research Centre, Faculty of Health Sciences, University of Johannesburg, P.O. Box 17011, Doornfontein 2028, South Africa; ericaniogo@gmail.com (E.C.A.); blassang@uj.ac.za (B.P.G.)

**Keywords:** P-glycoprotein, multidrug resistance, photodynamic therapy, apoptosis

## Abstract

Multidrug resistance (MDR) has posed a significant threat to cancer treatment and has led to the emergence of a new therapeutic regime of photodynamic therapy (PDT) to curb the menace. The PDT modality employs a photosensitiser (PS), excited at a specific wavelength of light to kill cancer cells. In the present study, we used a zinc phthalocyanine tetrasulfonic acid PS to mediate the photodynamic killing of MCF-7 cells overexpressed with P-glycoprotein (P-gp) and investigate the response to cell death induction. After photodynamic treatment, MCF-7 cells undergo cell death, and indicators like Annexin V/PI staining, DNA fragmentation, and measurement of apoptotic protein expression were investigated. Results showed increased externalisation of phosphatidylserine protein, measured as a percentage in flow cytometry indicative of apoptotic induction. This expression was significant (*p* < 0.006) for the untreated control cells, and there was no detection of DNA fragments after a laser fluence of 20 J/cm^2^. In addition, a statistically significant difference (*p* < 0.05) was seen in caspase 8 activity and Bax protein expression. These findings were indicative of apoptotic induction and thus seem to represent the extrinsic apoptotic pathway. This study shows the role of PDT in the treatment of a resistant phenotype breast cancer.

## 1. Introduction

Breast cancer continues to impact women and is the leading cause of global cancer incidence, with 2.3 million new cases annually [1]. Mammographic screening, chemotherapy, and adjuvant hormonal therapies have improved the survival rate of breast cancer. Yet treatment setbacks like tumour relapse and resistance continue to increase the burden on patients as well as lead to an enormous economic impact [2].

Proteins like P-gp, multidrug-resistant, and breast-cancer-resistant proteins have been identified as drug-resistant proteins. Their increased expression can transform the cell phenotype to a resistant variant [3,4]. Ideally, cell death occurs through a regulated process called apoptosis, to maintain normal tissue homeostasis. When this process is evaded, cancer ensues and increases its virulence [5]. Apoptosis is the evolutionarily conserved cell death process that activates cysteine-aspartic proteases (caspases) and the death receptor pathway [6]. Hence, there is a need for novel technologies in cancer treatment to improve outcomes and manage tumour resistance. Photodynamic therapy (PDT) combines the interaction of a photosensitizer (PS) and light of appropriate wavelengths to generate a highly reactive oxygen-related species that destroys tumour cells [7,8]. This emerging procedure has the advantage of preferential destruction of tumour cells; thus, its suggestion is a remedial therapy procedure for multidrug-resistant tumours [9,10]. PDT is a complex biochemical and photobiological reaction network, and it is essential to emphasise its usefulness in the mediation of resistant cancer death.

Understanding apoptosis and its regulation in the dynamic nature of PDT is still nascent and, if well understood, will lead to cancer control. In this study, we demonstrated the effect of zinc phthalocyanine tetrasulfonic acid (ZnPcS4)-mediated PDT on apoptotic induction of MCF-7 cells with increased P-glycoprotein expression. We showed that PDT could induce apoptosis by activating caspase 8 to mediate the desired killing effect on drug-resistant MCF-7 cells, depending on the light dose applied. 

## 2. Materials and Methods

### 2.1. Cell Culture Preparation

The human breast cancer cell line (MCF-7; ATCC HTB-22) with the lot number (60731981), purchased from American Type Culture Collection (ATCC), and increased P-gp expressing MCF-7 cells, otherwise referred to as MCF-7/DOX cells, were established in our laboratory [11] and used for this study. The antibodies used is listed in Table S1. The cells were maintained in Dulbecco’s Modified Eagle’s Medium (DMEM; D5796), supplemented with 10% Foetal Bovine Serum (FBS, 10499-044; Gibco), 1% Penicillin-Streptomycin (P4333), and 1% Amphotericin B (A2942) in an 85% humidified atmosphere at 37 °C and 5% CO_2_. Aseptic cell culture procedures were followed to grow the cells in T175 culture flasks (CR/431080) and passaged once 80% confluent. Cells were seeded at 5 × 10^5^ cells/cm^2^ concentration in 35 mm culture dishes and allowed for 4 h to attach before being used for experiments. For analysis of cells on a 96-well plate, following treatments on the culture plates, the cells were detached, collected, and seeded at a concentration of 2 × 10^4^ cells/90 µL per well. 

### 2.2. Photodynamic Treatment

The photodynamic therapy experiment was performed using a 681.0 diode laser with 200 Mw/cm^2^ power output and cells seeded at 5 × 10^5^ cells per 35 mm culture dishes. In our previous phototoxicity study, we established that a 45 µM concentration of ZnPcS4 in a combination of laser light of 20 J/cm^2^ caused a 50% decrease of MCF-7/DOX cell viability [11]. Hence, we used 45 µM concentration across different fluencies of laser light for mechanistic studies and determination of cell death following PDT [11]. The 45 µM of ZnPcS4 was added to MCF-7/DOX cells and incubated at 37 °C overnight. After that, cells were washed with phosphate-buffered saline (PBS) and irradiated at 5, 10, and 20 J/cm^2^ fluencies. After PDT treatment, cells were washed with hanks balanced salt solution (HBSS) and fresh media added and were kept at a 5% CO_2_ humidified atmosphere of 37 °C for 24 h before cellular response assessment. Light treatment without PS at 5, 10, and 20 J/cm^2^ was used as the laser control, and PS alone at 45 µM concentration without light was used as the dark control. The untreated control cells, treated with neither PS (ZnPcS4) nor light, were compared with the treated groups.

### 2.3. Annexin V/Propidium Iodide (PI) Flow Cytometric Analysis of Cell Death

A fluorescein Isothiocyanate (FITC) Annexin V/PI Apoptosis detection kit II (BD Pharmingen^TM^: 556570) was used to detect apoptosis in MCF-7/DOX cells. After PDT treatments, cells were harvested with trypLE^TM^ (Gibco 12563-029) and washed twice with cold PBS by centrifugation at 400× *g* in a 5 mL flow cytometer test tube before being suspended in 200 µL binding solution. After that, the cells were stained with a mixture of 5 µL of FITC Annexin V and 5 µL of PI. The tube was incubated in the dark at room temperature for 15 min. The mixture was analysed using a BD Accuri™ C6 flow cytometer (BD Biosciences, Franklin Lakes, NJ, USA).

### 2.4. Enzyme-Linked Immunosorbent Assay (ELISA) for Bcl-2 and Bax Proteins Detection

ELISA was used to detect and quantify the expression of Bcl-2 and Bax apoptotic proteins involved in the cell death pathway. The human Simple-Step Bcl-2 ELISA Kit (ab202411) and human Simple-Step Bax ELISA Kit (ab199080) from Abcam (Waltham, MA, USA) and a concentration of 2 × 10^4^ cells/90 µL per well were used for the assay. The kits measured the quantitative expression of the proteins in human cells using affinity tag labelled antibody and reporter conjugated detector antibody mix (capture antibody/analyte/detector antibody) on the 96-well plates. The treated MCF-7/DOX cells used as samples were prepared by centrifuging the cells at 500× *g* for 5 min at 4 °C. The pellets were solubilised in chilled 1× cell extraction buffer provided in the kit and incubated in ice for 20 min. After that, the solution was centrifuged at 18,000× *g* for 20 min. The pellets were discarded, and the supernatant was used as a sample. An equal volume (50 µL) of pieces and antibody cocktail mix was added to the appropriate wells on a 96-well plate for the assay measurement. The plate was incubated for 1 h at room temperature on a plate shaker set at 400× *g*. After that, wells were washed three times with 350 µL of 1× wash buffer (provided in the kit), 100 µL of Tetramethylbenzidine (TMB) substrate was added, and the mixture was incubated for 10 min in the dark. After the incubation, 100 µL of stop solution (provided in the kit) was added to each well and agitated for 1 min before the optical density measurement at 450 nm (Perkin-Elmer, Victor Nino^TM^ multimode plate reader, Pontyclun, UK).

### 2.5. Enzyme-Linked Immunosorbent Assay for Cytochrome c Protein Detection

The cytochrome c ELISA kit (Cat # KH01051, Thermo Fisher Scientific, Braamfontein, JHB, SA) was used to detect and quantify the level of cytosolic cytochrome c expression. The kit is provided with an affinity tag antibody and a reporter conjugated detector complex (capture antibody/analyte/detector antibody) immobilised on the 96-well plates. The treated MCF-7/DOX cells used as samples were prepared by centrifuging the cells at 500× *g* for 5 min at 4 °C. The cells were lysed in cell extraction buffer provided by the kit for 30 min on ice. After centrifugation at 13,000× *g* for 10 min, the supernatant was collected and used as a sample. The assay was performed by mixing equal volume (100 µL) of samples and biotin conjugate solution added on a 96-well plate and incubated for 1 h at room temperature on a plate shaker set at 400× *g*. After that, each well was washed three times with 350 µL of 1× wash buffer (provided in the kit), and 100 µL of 1× streptavidin-horseradish peroxidase (HRP) solution was added and incubated for 30 min. The plate was washed with 1× wash solution, 100 µL of stabilised chromogen was added, and the mixture was incubated for 30 min in the dark. Next, 100 µL of stop solution (provided in the kit) was added to each well before 1 min shaking and an optical density reading at 450 nm (Perkin-Elmer, Victor Nino^TM^ multimode plate reader, Pontyclun, UK).

### 2.6. Fluorometric Measurement of Caspase 8 and 9

This multiplex assay kit (ab219915) from Abcam provides a simple and convenient tool for quantifying caspase 8 and caspase 9 activity in cells undergoing apoptosis. Briefly, the cell pellet in culture media was added at a concentration of 2 × 10^4^ cells/90 µL per well in a black poly-d-lysine coated plate. The solution was mixed with 100 µL of each caspase assay loading solution, and the plate was incubated for 60 min at room temperature, protected from light. After that, a Perkin-Elmer, Victor Nino^TM^ multimode plate reader (Pontyclun, UK) was used to monitor the fluorescence at the following specific wavelengths: caspase 8:490 nm excitation/525 nm emission, and caspase 9:370 nm excitation/450 nm emission. The fluorescent intensity was determined using the relative fluorescence unit (RFU).

### 2.7. DNA Fragmentation Analysis (Gel Electrophoresis)

This is a semi-quantitative method to support the measure of apoptotic induction. It detects the internucleosomal DNA fragmentation in an apoptotic cell. This assay was conducted using a DNA fragmentation kit (ab66090; Abcam^®^). Treated cells were lysed with 35 µL of Tris-Ethylenediaminetetraacetic acid (TE) lysis buffer before adding 5 µL enzyme A solution, and the mixture was incubated at 37 °C for 10 min. Enzyme B (5 µL) was added, and the mixture was further incubated at 50 °C overnight. After that, 5 µL of ammonium acetate solution and 50 µL of isopropanol were added and mixed, and the mixture was kept at −20 °C for 10 min. The mixture was centrifuged at 400× *g* for 10 min to precipitate the DNA, 0.5 mL of 70% ethanol was added to wash, and the resultant pellet was mixed with 30 µL DNA suspension buffer. The suspension containing the DNA and molecular weight size marker (Bio-33053 HyperLadder^TM^ 1 kb Bioline) was mixed with 20 µL of Tris-acetate EDTA buffer. Electrophoresis was done using 1.2% agarose gel containing 0.5 µg/mL ethidium bromide, and the stained gel was visualised by the ChemiDoc MP imaging system (Bio-Rad, Hercules, CA, USA). 

### 2.8. Immunofluorescence of p53 Protein 

The immunofluorescent expression of wild-type p53 apoptotic protein was detected using a Carl Zeiss Axio Observer ZI (Jena, Germany). The MCF-7/DOX cells were seeded at 1 × 10^5^ on a coverslip, treated, and allowed to incubate overnight. The following morning, cells were fixed with 4% paraformaldehyde, permeabilised with 0.1% Triton X-100 in PBS, and blocked for non-specific binding with 10% bovine serum albumin (BSA) in PBS. The immunostaining was performed with primary monoclonal antibodies (1:100, anti-p53; CBL404 Merck company) overnight, followed by washing three times with PBS and incubation with secondary fluorescent-labelled antibody (1:500, Goat Anti-mouse (FITC), ab97050, Abcam) for 1 h at room temperature. Next, the nuclei of the cells were counterstained with 1 µg/mL 4′,6-diamidino-2-phenylindole (DAPI), and the coverslip was mounted for protein expression observation using a Carl Zeiss Axio Observer ZI (Jena, Germany). 

### 2.9. Western Blot Analysis of p53 Protein

The BCA protein assay reagent (Thermo Fisher Scientific, Rockford, IL, USA) was used to measure the concentration in cell lysate. The cell lysates were diluted in RIPA buffer to the gel-loading protein concentration (2.5 µg/µL) to mitigate the discrepancy of cell density, further mixed with an equal volume of sample buffer (0.125 M Tris/HCl pH 6.8, 10% glycerol, 4% sodium dodecyl sulphate (SDS), 0.25 M dithioerythritol (DTT)), and heated for 5 min at 110 °C. The SDS-polyacrylamide gel electrophoresis (SDS-PAGE) was conducted using a Bio-Rad (Hercules, CA, USA) electrophoresis machine. The proteins were transferred to a polyvinylidene difluoride (PVDF) membrane immunoblot (Bio-Rad Cat: 162-0177) overnight at 20 V, using a Semi-dry blotter (Sigma-B2529). Immunostaining was done with 5% BSA in tris-buffered saline (TBS) for 30 min for blocking, followed by primary antibody incubation with respective antibodies (1:100, anti-p53; CBL404 Merck company; 1:100, and glyceraldehyde-3-phosphate dehydrogenase (GAPDH) Cat # MA5-15738, Invitrogen) for 1 h. The TBS solution containing 0.1% Tween-20 was used to wash the membrane three times (5 min) before further incubation with secondary antibody conjugated with horseradish peroxidase (Goat anti-mouse HRP, Cat # SC-2005, Santa Cruz Biotechnology) for 2 h. Next, the membrane was rinsed three times with TBS and incubated with an equal volume of enhanced chemiluminescence (ECL) western blotting detection reagent (Amersham Cat no: RPN2209) for 5 min in the dark for band development and visualisation using the ChemiDoc MP imaging system (Bio-Rad, USA).

### 2.10. Statistical Analysis

The experiments were performed in duplicate in at least four independent repeats (*n* = 4). The untreated control cells were cells not treated with ZnPcS4 or laser light and were used to compare the treated cells in one-way ANOVA statistical analysis (Dunnett test) to determine the difference and significance using SigmaPlot version 13.0. Data were represented as mean ± standard error of the mean (SEM). The statistical significance was calculated as a *p*-value less than 0.05 (*), 0.01 (**), and 0.001 (***).

## 3. Results

### 3.1. Annexin V/PI Staining

**Figure 1:**
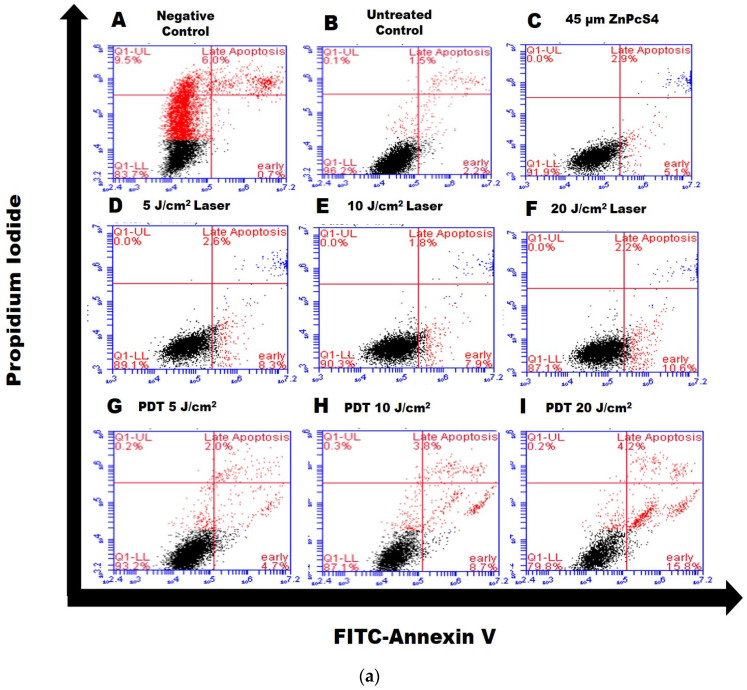

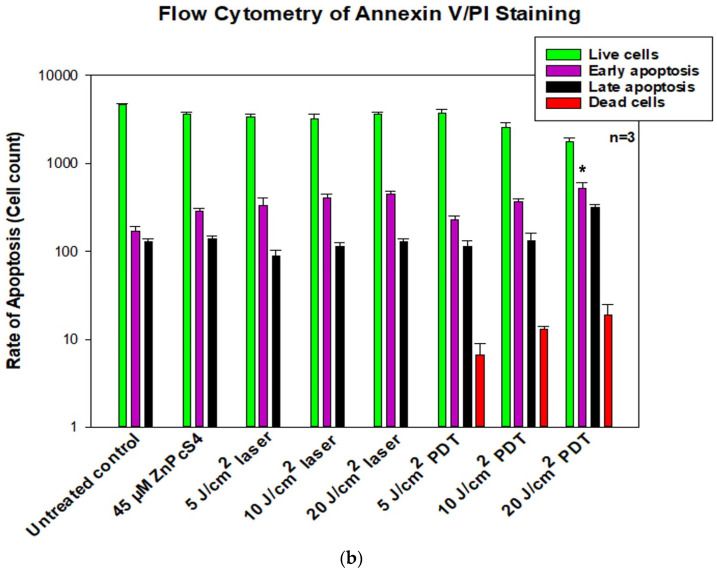
(**a**) Quadrant distribution of MCF-7/DOX cells stained with Annexin V/FITC and PI undergoing apoptosis, dead cells, late apoptosis, live cells, early apoptosis. The MCF-7/DOX control cells used were treated with hydrogen peroxide to induce death (image **A**), untreated control cells (image **B**), PS alone control (image **C**), and laser control difference of 5, 10, and 20 J/cm^2^ fluencies for images (**D**–**F**), respectively. The MCF-7/DOX PDT treated cells are represented in pictures (**G**–**I**) for 5, 10, and 20 J/cm^2^ fluencies, respectively. (**b**) ) Cell apoptosis was analyzed using flow cytometry after 24 h post-treatment for controls, 5, 10, and 20 J/cm^2^ treatment groups. There was a statistically significant difference * *p* < 0.06 in the rate of cells undergoing early apoptosis between the untreated control and 20 J/cm^2^ treated groups. Data are presented as mean ± standard deviation in the representative graphs of different apoptotic phase quadrants of flow cytometry (Figure 1a).

This assay helps to determine the percentage of apoptotic cell death induced by ZnPcS4-PDT in MCF-7/DOX cells. The flow cytometry results show different phases of apoptosis in quadrants (Figure 1a). The effects of laser, PS, and untreated controls were statistically analyzed, together with the experimental cell groups shown in Figure 1b. The data showed an increase in the number of cells undergoing early apoptosis in the 20 J/cm^2^ PDT treatment group. The ZnPcS4-induced apoptotic response depended on the laser fluencies applied, as there was a gradual decline and increase in the number of live and apoptotic cells, respectively (Figure 1a,b). The early apoptotic cells at 20 J/cm^2^ were significant (*p* < 0.006) compared to the untreated control group. 

The rate of late apoptotic cell counts was reduced. There was no difference between the untreated MCF-7/DOX control cells and other experimental PDT groups (5, 10, and 20 J/cm^2^), including PS alone and laser controls. Apoptosis is a time-dependent process, and there was an increase in the early apoptotic cell counts but not in the late stage. The untreated control cells were used to statistically compare the results of cells undergoing apoptotic (early and late) cell death. In the PDT experimental groups, the presence of dead cells was also observed (Figure 1b), unlike the laser and PS alone controls. 

### 3.2. ELISA Expression of Bcl-2, Bax, and Cytochrome c Proteins

An enzyme-linked immunosorbent assay measured the photodamage and activation of Bax/Bcl-2 and cytochrome c apoptotic proteins. The results showed increased Bax relative to Bcl-2 protein expression (Table 1). The amount of Bax expressed in control cells was 1.73 ng/mL, and the experimental group amounts were 2.31, 2.11, and 2.84 ng/mL for 5, 10, and 20 J/cm^2^, respectively. The results for Bcl-2 expression showed a significant (*p* < 0.05) decrease to 1.21 ng/mL for 20 J/cm^2^ compared to 1.62 ng/mL in the untreated control. We found no significant release of cytochrome c among the treated 5, 10, and 20 J/cm^2^ groups, indicating that PDT did not induce cytochrome c release. 

### 3.3. DNA Fragmentation Assay

**Figure 2:**
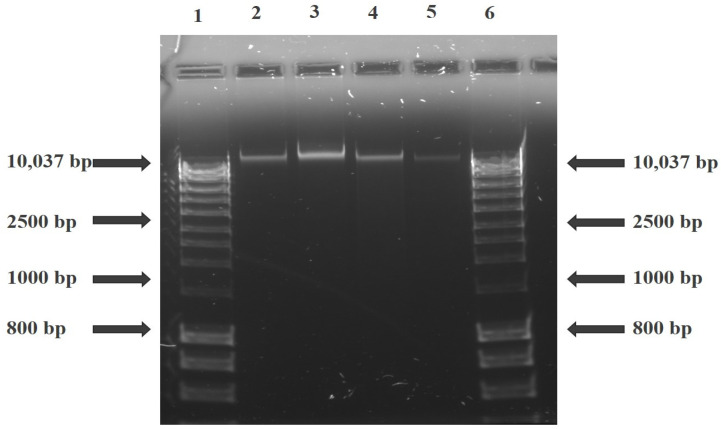
Agarose gel electrophoresis of MCF-7/DOX cell extracted DNA after ZnPcS4—PDT treatment. Lanes 1 and 6 are loaded with DNA hyper-ladder, and Lanes 2, 3, 4, and 5 with the untreated control, 5, 10, and 20 J/cm^2^ fluencies, respectively.

The DNA fragmentation assay was used to analyse the level of DNA damage during the apoptosis induction. The results showed no fragmentation of the DNA after ZnPcS4-induced PDT with different laser fluencies on MCF-7/DOX cells. Figure 2 shows that the total amount of DNA in the control (Lane 2) and treated experimental groups (Lanes 3, 4, and 5 representing 5, 10 and 20 J/cm^2^, respectively) did not show a typical ladder formation oligonucleosomal fragmentation. Lanes 1 and 6 were loaded with DNA hyper-ladder used as a reference to estimate the DNA fragment sizes (Figure S1).

### 3.4. Fluorometric Measurement of Caspase 8 and 9

**Figure 3:**
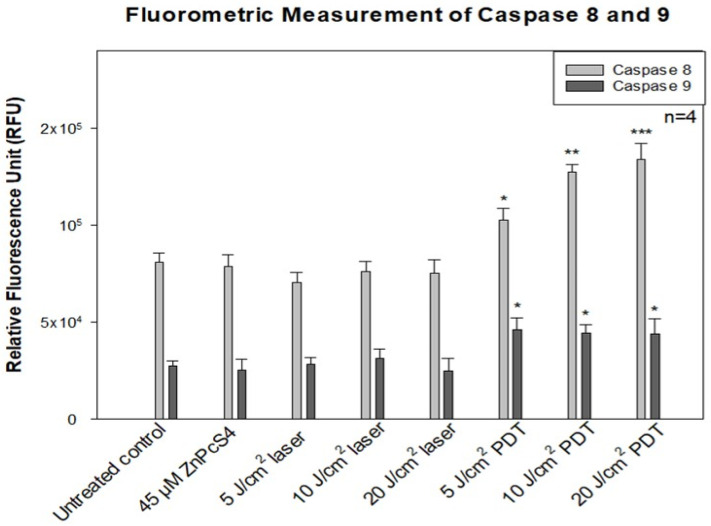
Fluorometric measurement of caspase 8 and 9 activities in MCF-7/DOX cells. The cellular photodamage induced by ZnPcS4-PDT significantly increased with * *p* < 0.01, ** *p* < 0.001, and *** *p* < 0.0006 the activities of caspase 8 in a fluence-dependent manner for 5, 10, and 20 J/cm^2^, respectively. Similarly, caspase 9 activity showed a significant (*p* < 0.05) difference in all experimental groups compared to the untreated control. PS dark toxicity and laser controls showed no difference in comparison with the untreated control.

The expression activities of initiator caspase 8 (extrinsic) and caspase 9 (intrinsic) were measured using the fluorometric indicators, Rhodamine 110 (R110, green fluorescence) and 7-amino-4-methyl coumarin (AMC, blue fluorescence), respectively, in MCF-7/DOX cells undergoing apoptosis. After ZnPcS4-PDT, a significant difference was recorded with caspase 8 as the fluence of the laser treatment increased. The caspase 8 activity had a dose-dependent increase with the *p* values, *p* < 0.01, *p* < 0.001, and *p* < 0.0006 for 5, 10, and 20 J/cm^2^ treated groups compared to the untreated control. This observation was not seen in cells treated with PS alone and laser controls (Figure 3). In caspase 9 activity, there was no significant correlation among the experimental treated groups (5, 10, and 20 J/cm^2^), but when analyzed with the untreated control, a statistical difference (*p* < 0.05) was seen. These results showed that ZnPcS4-induced PDT increased caspase 8 activity, followed by a slight caspase 9 activity in MCF-7/DOX cells. In the PS dark toxicity experiment and laser control of 5, 10, and 20 J/cm^2^ fluencies, there was no significance in caspase 8 and 9 activities compared with the untreated control.

### 3.5. Immunofluorescence of p53

**Figure 4:**
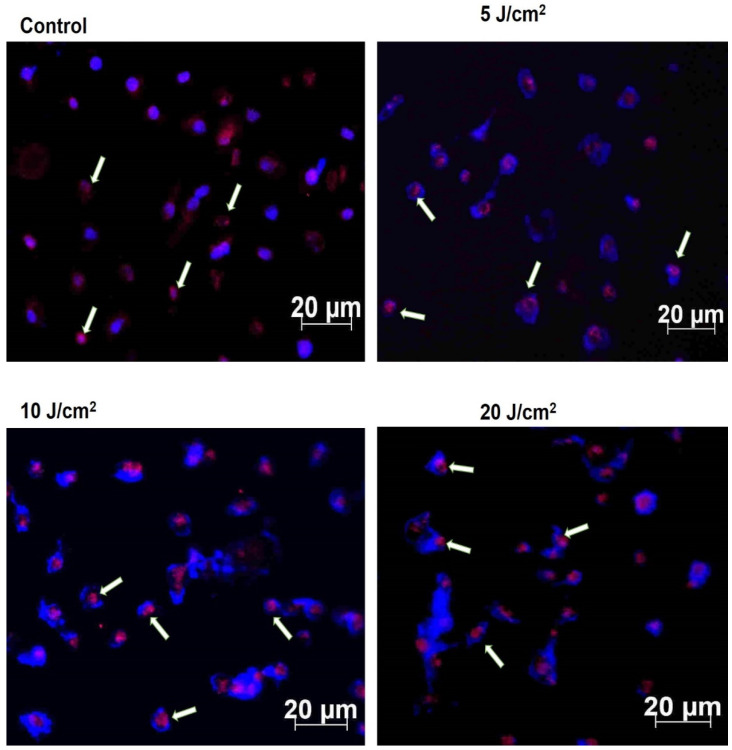
Evaluation of p53 expression in MCF-7/DOX cells. The p53 protein in pink is shown against the blue nuclear matrix in different fluencies of light at 5, 10, and 20 J/cm^2^ and the untreated control.

The central role of tumour suppressor protein p53 in apoptosis induction after ZnPcS4-PDT was analysed using the immunofluorescence protein expression technique. In response to the ZnPcS4—PDT, the p53 expression (pink) was seen within the nuclear matrix (blue) of the MCF-7/DOX cells (Figure 4). There was the detection of p53 protein expression among the experimental treated groups (5, 10, and 20 J/cm^2^), which mediates the response of p53 to the radiation, DNA damage, oxidative stress, and hypoxia stimuli after ZnPcS4-PDT treatment. 

### 3.6. Western Blotting Analysis of p53 Protein

**Figure 5:**
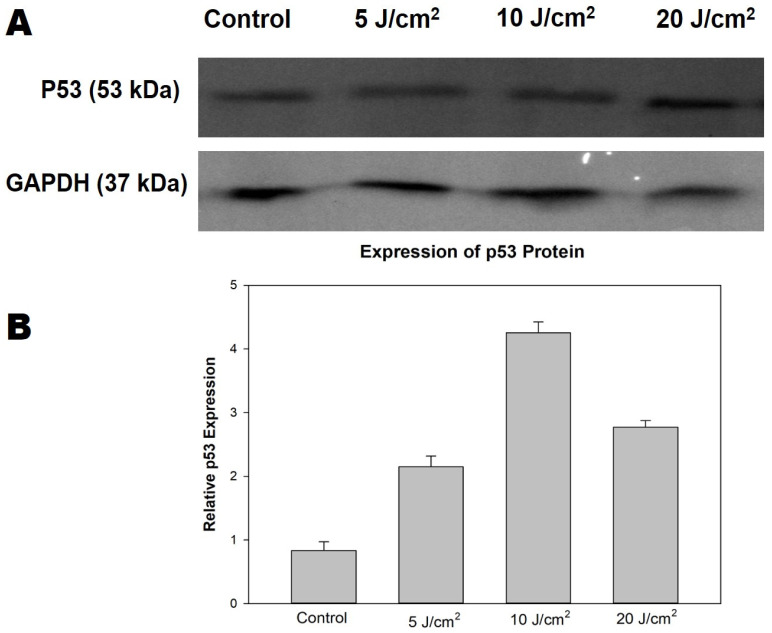
The ZnPcS4-PDT induced expression of p53 on MCF-7/DOX cells (**A**). Protein expression in bands on SDS-Polyacrylamide gel detected using ChemiDoc MP imaging system (Bio-Rad, USA). The protein bands were quantified using ImageJ software and represented in the histogram (**B**) based on the relative expression value of the grayscale bands corresponding to p53 compared to the housekeeping gene (GAPDH).

The specificity of the tumour suppressor wildtype p53 protein was measured using the immunoblotting technique in MCF-7/DOX cells undergoing apoptosis. The suppressor protein plays a crucial role in response to oxidative stress or cellular photodamage induced by PDT stress. There was a detectable level of nuclear p53 protein (53 kDa) expression in the grey bands (Figure 5A). The observation of p53 expression among the treatment groups suggests its role in tumour suppression after ZnPcS4-PDT treatment. Each band on the immunoblot was normalised with the housekeeping (GAPDH) gene to determine the relative expression, as shown in Figure 5B. 

## 4. Discussion

Multidrug resistance is one of the main reasons why cancers prove challenging to treat. Most cancer cells with increased P-gp phenotype are resistant to chemotherapeutic treatment due to the P-gp membrane protein, which constantly extrudes harmful substances out of the cell [12,13,14,15]. The findings of the present study revealed that PDT on MCF-7/DOX cells with increased p-gp phenotype triggered the expression of caspase 8, Bax, and p53 proteins in the process of cell death. The MCF-7/DOX cells used in the study were isolated through repeated exposure to Doxorubicin (DOX) [11]. The isolated resistant phenotype cells were used as a model for subsequent photodynamic therapy investigation of drug action and treatment. At present, there has been little research about the specificity of cell damage and mode of death following PDT on resistant MCF-7 cells. Here, the molecular response of MCF-7/DOX cells to death induction following PDT was evaluated. Apoptotic cell death indicator protein phosphatidylserine was first measured using Annexin V/PI staining. The results showed an increased percentage of apoptotic cells after ZnPcS4-induced PDT, which depended on the laser fluence used (20 J/cm^2^), with a significance of *p* < 0.0006 compared to the untreated control. The extent to which apoptosis or necrotic cell death occurs depends on factors like the intracellular localization of the photosensitizers, light dose, and the target cell’s specific properties [16,17]. The laser controls and PS alone treatment groups showed no significance in the apoptosis induction, unlike the PDT groups. PDT with increased energy favours the apoptotic pathway by activating procaspase 9, which surges nuclear signal transduction, chromatin condensation, and fragmentation [17,18]. 

The process of apoptosis is initiated via two major pathways: extrinsic (or death receptor) and intrinsic (or mitochondrial) [19]. The latter is triggered by the Bcl-2 family proteins and the release of cytochrome c from the mitochondria. Hence, we examined the expression of proapoptotic Bax and antiapoptotic Bcl-2 proteins on the apoptotic induction after ZnPcS4-PDT treatment. Our results showed an increase in the Bax to Bcl-2 expression, with no significant release of cytochrome c, which implies minimal photodamage to the mitochondria. This might result from less accumulation of ZnPcS4 in the mitochondria [11] and, as such, reduced mitochondrial photodamage. However, Plonka and colleagues’ contrary findings showed a decrease in Bax to Bcl-2 protein on MCF-7 and T-47D tumour cells treated with Photolon-induced PDT [20]. Another report by Chiu et al. suggests that Pc4-mediated PDT induced Bax translocation from the cytosol to mitochondria for the intrinsic apoptotic pathway activation in MCF-7c3 cells [21]. DNA damage and fragmentation are the hallmarks of apoptosis, and in the present study, we measured the DNA fragmentation characteristics during apoptotic induction on the treated cells. There was no enzymatic activity of endonuclease proteins, and thus no fragmentation process was observed (Figure 2). This confirms the non-expression of the CASP-3 gene in MCF-7 cells due to exon deletion of the CASP-3 gene that abrogates its mRNA translation [22,23]. Expression of caspase 3 contributes to membrane blebbing and cleavage of caspase-activated DNase inhibitor, which leads to the typical DNA fragmentation pattern observed during apoptosis [23]. Despite this, other breast cancer in vitro studies have shown the involvement of dysregulated caspase gene activity in drug-resistant cells restored through plasmid procaspase 3 cDNA transfection [24,25]. 

The measurement of initiator caspase 8 (extrinsic) and 9 (intrinsic) proteins showed increased caspase 8 activity after ZnPcS4-mediated PDT. This implies the activation of the death receptor ligand in the cell membrane, which contributes to apoptosis. Studies by El-Hussein et al. and Tynga et al. with Zinc Sulfo Phthalocyanine PS on MCF-7 cells showed apoptosis through the mitochondria-mediated intrinsic pathway of caspase 9 and 3 activations [26,27]. Contrary to their findings, this study observed that the apoptotic cell death pathway induced was due to extrinsic induction through phosphatidylserine translocation and death receptor ligand activation, which increases the caspase 8 activity. It should also be taken into consideration that PDT induction of cell death depends on many factors, like PS concentration, localization, and the light dose used for irradiation, among others [16,17]. The energy supply for photodynamic reaction with biomolecules has been found to favour apoptosis in cancer cells by activating the procaspase 9 enzyme that surges nuclear signal transduction, chromatin condensation, and fragmentation [17,18]. 

Evidence has shown that PDT-mediated apoptosis in mouse embryonic fibroblast cells is modulated by p53 protein [28]. Hence, we measured the wildtype p53 expression by immunofluorescence and western blotting analysis to assess its role in inducing the apoptotic process. The p53 protein plays a central role in cellular DNA damage responses, and any mutation in the protein can lead to cell survival [29]. Our results showed p53 expression in MCF-7/DOX cells, which might contribute to triggering apoptosis after PDT. The result of a p53 increase in laser fluence of 10 J/cm^2^ was observed (Figure S2), indicating the efficacy of moderate light exposure for a better yield of high therapeutic response. A similar study using hypericin-mediated PDT on resistant colon adenocarcinoma cells (HT-29) suggests the dual involvement of p53 in both apoptotic response and survival processes [30]. In the PDT field, the dependence of cell-killing of p53 is still much debated, since research proposing both reliance and no correlation has been published. Herein, we resolved that the exact mechanism and correlation of p53 with apoptosis is not well understood. However, we hypothesize that the stress response of MCF-7/DOX cells induced by DNA damage, radiation, oxidative stress during the process of repeated chemotherapy, and the hypoxic activities of PDT treatment might have altered the upregulation of p53 to trigger cellular apoptosis. Hence, an in-depth experiment on gene expression and transcription protein activity after PDT is necessary to elucidate and offer more explanations. 

## 5. Conclusions

In conclusion, this study provided evidence that ZnPcS4-PDT induced apoptotic induction on MCF-7/DOX cells. The apoptosis induced was not mitochondrial-dependent due to low cytochrome c release, which supported the preferential localization of ZnPcS4 PS at the lysosome rather than mitochondria [11]. The involvement of caspase 8 is more prominent in the apoptotic process of ZnPcS4-PDT on MCF-7/DOX cells. Thus follows the receptor activation of the extrinsic pathway, as evidenced in the affinity of the Annexin V to the externalization of phosphatidylserine, a death receptor protein. The findings presented here suggest that PDT may act as a helpful treatment modality for resistant cancer types.

## Figures and Tables

**Table 1:** The enzyme immunoassay measurement of Bax, Bcl-2 and cytochrome c proteins showed increased Bax expression compared to Bcl-2 and cytochrome c release across all the experimental groups. Significance differences were presented as * *p* < 0.05, ** *p* < 0.01.

Parameter	Control (ng/mL)	5 J/cm^2^ (ng/mL)	10 J/cm^2^ (ng/mL)	20 J/cm^2^ (ng/mL)
Bax	1.730	2.311	2.112	2.845 **
Bcl-2	1.620	2.050	1.570	1.210 *
Cytochrome c	0.223	0.230	0.228	0.226

## Supplementary Materials

The following are available online. Figure S1: the original image of DNA fragmentation gel electrophoresis. Figure S2: original images of p53 and GAPDH expression on blot before cropping. Table S1: list of antibodies used in the study.

## Sample Availability

Cell samples used are available from the authors.
