# Supplementary material for: Response of MCF-7 Breast Cancer Cells Overexpressed with P-Glycoprotein to Apoptotic Induction after Photodynamic Therapy
Source: Molecules. 2021 Dec 6;26(23):7412. doi: 10.3390/molecules26237412 (PMC8658844; doi:10.3390/molecules26237412)
Supplement: Supplementary file 1 [file molecules-26-07412-s001.zip › molecules-1441668-supplementary.pdf]

RELATED INFORMATION

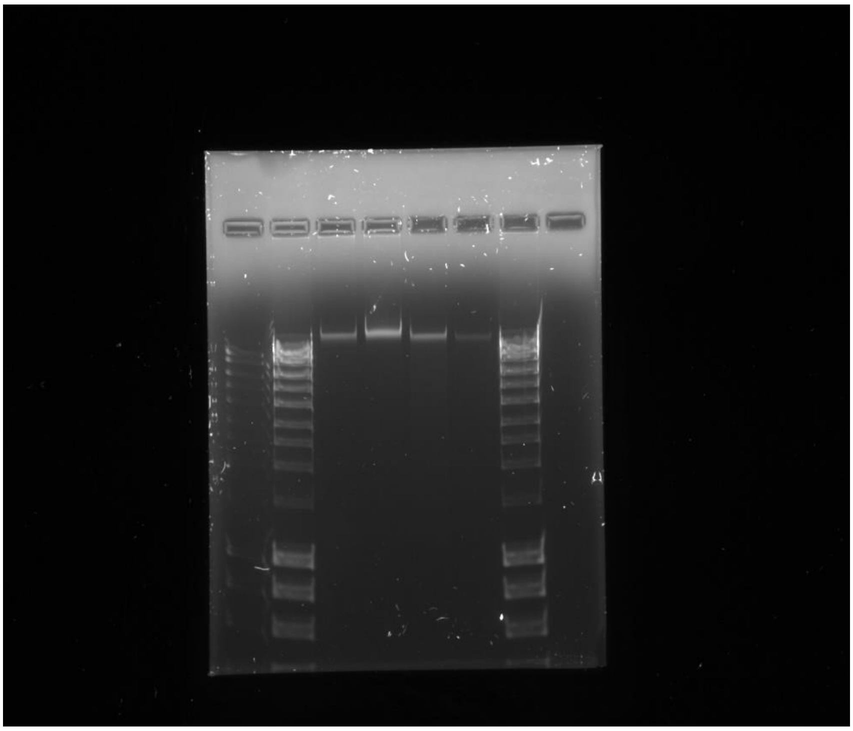

Figure S1. Original images of Gel Electrophoresis: The DNA extracted from ZnPcS4-mediated photodynamic therapy of the same concentration and different fluencies 5, 10, and 20 J/cm^2 were loaded Agarose gel electrophoresis. Lanes 1 and 6, were loaded with DNA hyper-ladder, and Lanes 2, 3, 4, and 5 with the untreated control, 5, 10, and 20 J/cm^2 fluencies, respectively.

A B

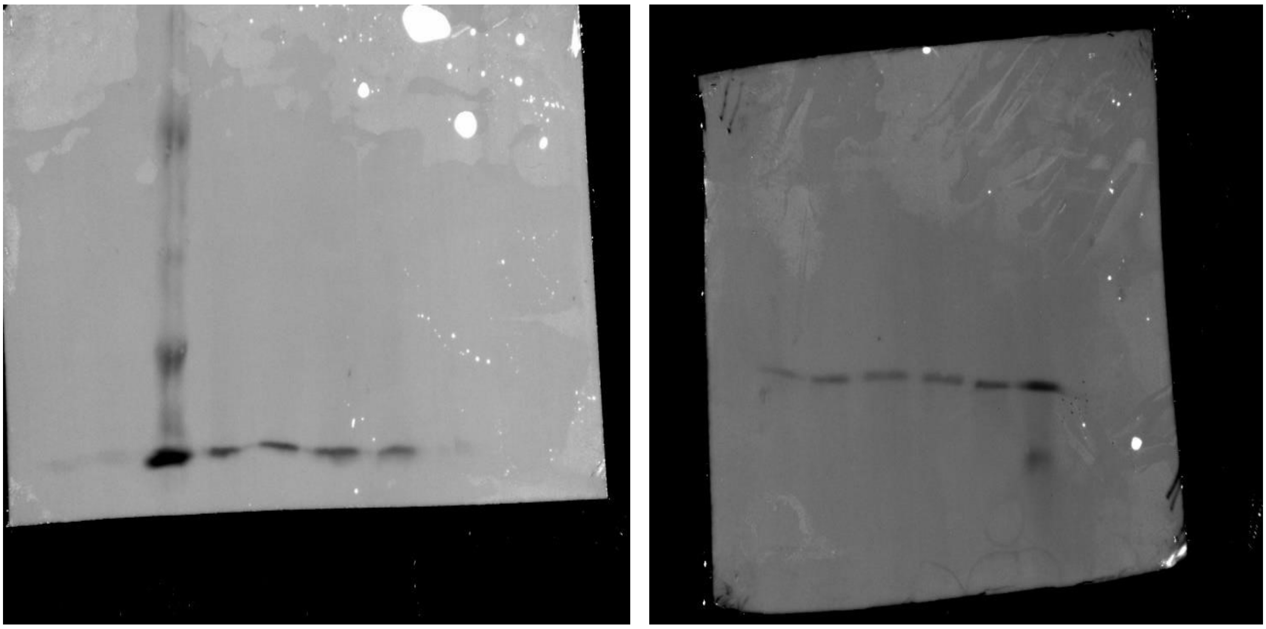

GAPDH expression p53 protein expression

Figure S2. Original images of western blots for p53 and GAPDH: The GAPDH expression (**image A**) was used as the housekeeping gene and (**image B**) is the expression of p53 protein. The lanes are loaded with protein ladder, followed by untreated control, ZnPcS4-mediated PDT treated by 5, 10, and 20 J/cm^2 respectively. Protein expression in bands on SDS-Polyacrylamide gel was detected using ChemiDoc MP imaging system (Bio-Rad, USA).

Table S1. List of antibodies used in the study.

**Antibody**	**Company**	**Cat. No.**
Anti-p53 antibody	Merck South Africa	CBL 404
Goat anti-mouse antibody	Abcam South Africa	Ab97050
Anti-glyceraldehyde-3-phosphate dehydrogenase (GAPDH)	Invitrogen South Africa	MA5-15738
Goat anti-mouse HRP	Santa Cruz Biotechnology	SC-2005
